# Artemether-lumefantrine co-administration with antiretrovirals: population pharmacokinetics and dosing implications

**DOI:** 10.1111/bcp.12529

**Published:** 2014-10-08

**Authors:** Richard M Hoglund, Pauline Byakika-Kibwika, Mohammed Lamorde, Concepta Merry, Michael Ashton, Warunee Hanpithakpong, Nicholas P J Day, Nicholas J White, Angela Äbelö, Joel Tarning

**Affiliations:** 1 Unit for Pharmacokinetics and Drug Metabolism, Sahlgrenska Academy at the University of GothenburgGothenburg, Sweden; 2 Department of Medicine, School of Medicine, Makerere University College of Health SciencesKampala, Uganda; 3 Infectious Diseases Institute, Makerere University College of Health SciencesKampala, Uganda; 4 Mahidol-Oxford Tropical Medicine Research Unit, Faculty of Tropical Medicine, Mahidol UniversityBangkok, Thailand; 5 Centre for Tropical Medicine, Nuffield Department of Medicine, University of OxfordOxford, UK

**Keywords:** artemether-lumefantrine, drug–drug interaction, efavirenz, lopinavir/ritonavir, nevirapine, population pharmacokinetics

## Abstract

**AIM:**

Drug–drug interactions between antimalarial and antiretroviral drugs may influence antimalarial treatment outcomes. The aim of this study was to investigate the potential drug–drug interactions between the antimalarial drugs, lumefantrine, artemether and their respective metabolites desbutyl-lumefantrine and dihydroartemisinin, and the HIV drugs efavirenz, nevirapine and lopinavir/ritonavir.

**METHOD:**

Data from two clinical studies, investigating the influence of the HIV drugs efavirenz, nevirapine and lopinavir/ritonavir on the pharmacokinetics of the antimalarial drugs lumefantrine, artemether and their respective metabolites, in HIV infected patients were pooled and analyzed using a non-linear mixed effects modelling approach.

**RESULTS:**

Efavirenz and nevirapine significantly decreased the terminal exposure to lumefantrine (decrease of 69.9% and 25.2%, respectively) while lopinavir/ritonavir substantially increased the exposure (increase of 439%). All antiretroviral drugs decreased the total exposure to dihydroartemisinin (decrease of 71.7%, 41.3% and 59.7% for efavirenz, nevirapine and ritonavir/lopinavir, respectively). Simulations suggest that a substantially increased artemether-lumefantrine dose is required to achieve equivalent exposures when co-administered with efavirenz (250% increase) and nevirapine (75% increase). When co-administered with lopinavir/ritonavir it is unclear if the increased lumefantrine exposure compensates adequately for the reduced dihydroartemisinin exposure and thus whether dose adjustment is required.

**CONCLUSION:**

There are substantial drug interactions between artemether-lumefantrine and efavirenz, nevirapine and ritonavir/lopinavir. Given the readily saturable absorption of lumefantrine, the dose adjustments predicted to be necessary will need to be evaluated prospectively in malaria-HIV co-infected patients.

**WHAT IS ALREADY KNOWN ABOUT THIS SUBJECT**

- The pharmacokinetic properties of artemether, lumefantrine and their active metabolites have been well described.
- Lumefantrine exhibits dose dependent absorption, which might result in additional complexity in dose optimization.
- It is known that an interaction between artemether-lumefantrine and efavirenz, nevirapine and lopinavir/ritonavir exists but the magnitude and thus clinical relevance of the interaction has been uncertain.

**WHAT THIS STUDY ADDS**

- The pharmacokinetics of the parent antimalarial drugs and their metabolites are affected substantially by concomitant treatment with HIV drugs. These interactions are likely to be therapeutically relevant.
- The study quantifies the individual contributions of the different drug–drug interactions.
- Increased dosing of artemether-lumefantrine is needed when combined with efavirenz or nevirapine. Whether dose adjustments are needed when combined with lopinavir/ritonavir remains uncertain.

## Introduction

Malaria and human immunodeficiency virus (HIV) are amongst the most widespread and important infectious diseases in humans. In 2012, there were approximately 207 million cases of malaria according to World Health Organization estimates resulting in 627 000 deaths [1]. In 2011, 34 million people were estimated to be infected with HIV [2]. HIV infection increases the risk of symptomatic and severe malaria and compounds the adverse effects of malaria in pregnancy [3,4]. Because of the geographical overlap, particularly in Africa, antimalarials and antiretroviral drugs are commonly taken together. The most widely used treatment for falciparum malaria is the artemisinin-based combination treatment artemether-lumefantrine, given on six dosing occasions over 3 days (four tablets at each occasion in the adult dose). Artemether is a rapidly acting drug with a terminal half-life of 1.5–2.0 h [5–8]. It is metabolized rapidly into its active metabolite dihydroartemisinin, which has a half-life of 0.8–2.1 h [5–7]. The cytochrome P450 (CYP) enzymes CYP2B6, CYP3A4/5, CYP2C9 and CYP2C19 are responsible for demethylation of artemether with a small contribution from CYP2A6 [9,10]. Dihydroartemisinin is then converted to inactive metabolites by glucuronidation via uridine diphosphoglucurosyltransferase (UGT) A1, UGT1A9 and UGT2B7 [11]. There is significant enzymatic auto-induction of artemether demethylation in the course of a standard 3 day treatment [12].

Lumefantrine has an elimination half-life of 2–4 days and is responsible for eliminating the residual parasites that remain in the blood after the 3 day course of artemether, thereby preventing recrudescence [13,14]. Lumefantrine is metabolized mainly by CYP3A4 into its active metabolite desbutyl-lumefantrine [15]. Desbutyl-lumefantrine exhibits an antimalarial effect [16], but the systemic exposure to lumefantrine is 85- to 300-fold higher than to its metabolite. The parent drug is therefore responsible for most of the clinical antimalarial activity [17,18]. Lumefantrine absorption is increased by co-administration with fats and exhibits dose-dependent absorption kinetics with decreasing oral absorption with increasing doses [13].

HIV infection is most commonly treated with a combination of two nucleoside reverse transcriptase inhibitors and either a protease inhibitor, a non-nucleoside reverse transcriptase inhibitor or an integrase inhibitor. The first line non-nucleoside reverse transcriptase inhibitors in most low to middle income countries are efavirenz and nevirapine. Efavirenz and nevirapine induce CYP3A4 and CYP2B6 [19–21]. Studies on the effects of nevirapine and efavirenz on the activity of P-glycoprotein provide contradictory conclusions [22–26]. Lopinavir is a protease inhibitor, co-formulated with the protease (and CYP3A4) inhibitor ritonavir. When co-administered, lopinavir and ritonavir inhibit CYP 3A4 and UGTs 1A1, 1A3, 1A4, 1A6, 1A9 and 2B7. In contrast lopinavir and ritonavir induce CYPs 1A2, 2B6, 2C9 and 2C19 [19,27,28]. *In vitro* studies suggest that lopinavir may have a direct antimalarial effect although results from clinical studies do not provide consistent findings [29–31].

Interactions between HIV and antimalarial drugs could affect the efficacy of antimalarial treatment. Underexposure would increase treatment failure rates and thereby increase the risk of antimalarial drug resistance whereas overexposure could result in toxicity. Several studies have identified an interaction between artemether-lumefantrine and efavirenz, nevirapine and lopinavir/ritonavir [31–36].

The aim of the present study was to characterize the pharmacokinetic properties of the antimalarial drugs artemether/dihydroartemisinin and lumefantrine/desbutyl-lumefantrine when administered alone and in combination with efavirenz, nevirapine or lopinavir/ritonavir in HIV-infected individuals without malaria and where appropriate suggest new treatment regimens for these drugs when used concomitantly.

## Methods

### Clinical studies

The two clinical studies were conducted at Mulago National Referral Hospital in Kampala, Uganda. Details of both clinical studies are described in detail elsewhere [35,36].

#### Study 1

Study 1 was a parallel group study in HIV-infected patients. Inclusion criteria were age >18 years, informed written consent and a confirmed HIV diagnosis. Exclusion criteria were anaemia (haemoglobin levels below 8 g dl^–1^), viral loads above 400 counts ml^−1^, malaria parasitaemia, abnormal liver or renal function, pregnancy, the use of substances which inhibit or induce CYP enzymes or P-glycoprotein, use of herbal medicines, prolonged QT interval, or any intercurrent illness. Ethics approval was received from the Uganda National HIV/AIDS Research Committee (ARC 056) and the Uganda National Council of Science and Technology (HS 195) and the study was registered with http://ClinicalTrials.gov (NCT 00619944).

#### Study 2

Study 2 was a three period, crossover study in HIV-infected patients. Inclusion and exclusion criteria were the same as for study 1 (i.e. age >18 years, informed written consent and a confirmed HIV diagnosis) except that there were no restrictions based on viral load, and patients with hypersensitivity to artemisinins, lumefantrine or halofantrine or any history of cardiac disease were excluded from the study. Ethics approval was received from the Uganda National HIV/AIDS Research Committee (ARC 059) and from the Uganda National Council of Science and Technology (HS 196). The study was registered at http://ClinicalTrials.gov (NCT00620438).

### Treatment and sampling

#### Study 1

Patients in the first arm were treated with 400/100 mg of lopinavir/ritonavir (Aluvia®, Abbott Laboratories, Abbott Park, Illinois, USA) and two nucleoside reverse transcriptase inhibitors (zidovudine and didanosine or tenofovir and emtricitabine, regional non-controlled procurement). The drugs were taken twice daily for at least 1 month before the start of the study. Patients in the second arm had not yet started their HIV therapy. At the initiation of the study all patients received a single dose of 80 mg artemether and 480 mg lumefantrine (four tablets) (Coartem®, Novartis Pharma AG, Basel, Switzerland; Batch number: F0660). Venous plasma samples were drawn at 1, 2, 4, 6, 8, 12, 24, 48 and 72 h post-dose.

#### Study 2

At the start of the study, all patients received a standard treatment of artemether and lumefantrine (80/480 mg twice daily for 3 days; Coartem®). Plasma samples were collected after the last dose at 1, 2, 4, 8, 12, 24, 48, 72, 96 and 120 h post-dosing. After 1 week of wash-out, patients started HIV treatment with either nevirapine (200 mg) or efavirenz (600 mg) combined with the nucleoside reverse transcriptase inhibitors zidovudine and lamivudine. Patients received HIV treatment alone for 1 month and then were given a standard six dose treatment of artemether and lumefantrine concomitantly. Plasma samples were again collected after the last artemether-lumefantrine dose.

All samples from the two studies were stored at −70°C until drug quantification.

### Drug analysis

Artemether/dihydroartemisinin and lumefantrine were quantified in plasma samples according to previously published methods [37,38]. Artemether and dihydroartemisinin were measured using solid phase extraction and liquid chromatography coupled with tandem mass spectrometric detection. The total coefficient of variation (%CV) was less than 6% and the lower limit of quantification was set to 1.4 ng ml^−1^. Lumefantrine was measured using solid phase extraction followed by liquid chromatography with ultraviolet detection. The total coefficient of variation was below 6% and the lower limit of quantification was set to 25 ng ml^−1^.

Desbutyl-lumefantrine was measured using liquid chromatography (Model 1200 system, Agilent Technologies) coupled with tandem mass spectrometric detection (API5000 triple-quadrupole system, AB Sciex). Desbutyl-lumefantrine and its analogue internal standard (C_28_H_28_CL_3_NO [39]) were extracted from plasma samples (100 μl) by protein precipitation followed by phospholipid removal (Hybrid SPE-Precipitation 96-wellplate, 575656-U, Supelco). Chromatographic separation was performed on a Halo Amide reversed C18 column (50 mm × 2.1 mm, i.d. 2.7 μm, 92812-407, Advanced Materials Technology) protected by a Halo Amide guard column (5 mm × 2.1 mm, i.d. 2.7 μm, 92812-107). A gradient program was used consisting of a mobile phase of (A) acetonitrile-ammonium acetate 2.5 mm pH 3.5 (30–70, v/v) and (B) acetonitrile-ammonium acetate 10 mm pH 3.5 (80–20, v/v); 0–2 min 100% B, 2.1–2.9 min 100% A, 3–5.4 min 100% B, at a flow rate of 0.5 ml min^−1^. Quantification was performed using electrospray ionization in the positive mode and multiple reaction monitoring for the transitions m/z 472.1–346.1 and 537.2–346.1 for desbutyl-lumefantrine and its internal standard, respectively. The bioanalytical assay was validated to cover the therapeutic range of 1.00 ng ml^−1^ to 769 ng ml^−1^. A quadratic regression with 1/x^2^ weighting was used for quantification. The method was validated according to FDA guidelines [40] and showed a high accuracy of 96.1–102% at all quality control concentrations (i.e. 2.86, 40.6 and 577 ng ml^−1^). The within-day and between-day precision (%CV) were 1.25–5.82% at all quality control levels, with an absolute recovery of approximately 70–80%. Validation of over-curve samples ensured accuracy and precision when diluting samples outside the calibration range. The method did not show any signs of severe ion suppression/enhancement. The lower limit of quantification was set to 1.0 ng ml^−1^.

### Pharmacokinetic analysis

Modelling and simulations were performed in nonmem v7.12 (Icon Development Solutions, Ellicott City, Maryland, USA). Model discrimination was based on the objective function value (OFV) which is proportional to −2 times the log-likelihood of the data [41]. A drop of at least 3.84 or 6.64 was deemed significant with significance levels of 0.05 and 0.01, respectively, when adding one parameter in a nested model. Pirana v2.6.0 was used to document the modelling process [42]. R v2.14.2 (the R Foundation for Statistical Computing, Vienna, Austria) and the package Xpose v4.3.5 [43] was used to perform model diagnostics. Perl-speaks-nonmem v3.5.3 was used for automation throughout the modelling process [44]. Data below the limit of quantification were initially omitted or modelled as categorical data (M3-method), if necessary [45]. Simulation-based diagnostics (i.e. fraction of simulated and observed data below the limit of quantification) were used to evaluate the impact of omitting such data.

All concentrations were converted into their natural logarithms. Parent drug and metabolite concentrations were fitted simultaneously. Both artemether and lumefantrine were assumed to be fully converted into their respective metabolite. One, two and three compartment disposition models were evaluated. An additive residual error on log-transformed data (essentially equivalent to an exponential error on normal scale data) was used.

Different absorption models were tested, including first-order absorption, first order absorption with lag time, zero order absorption, sequential zero and first order absorption, and a transit compartment absorption model with 1–10 fixed transit compartments [46].

Enzymatic auto-induction of artemether metabolism was evaluated using a sub-set of data in which artemether-lumefantrine was administered alone over 1 or 3 days. Auto-induction was modelled using a maturation-model with a maturation half-life fixed to a mean of literature values (i.e. 62 h) [47] and the maximum maturation were assumed to be 100%.

Between subject variability was added exponentially to each parameter. Box–Cox transformation of the variability was also tried to evaluate formally the distribution of the random effects. A fixed bioavailability of 100% for the population with an estimated between subject variability was evaluated.

Concomitant treatment with HIV drugs was added as a categorical covariate, one for each HIV treatment. The covariate was added in a stepwise manner with a forward criterion of *P* < 0.05 and a backward criterion of *P* < 0.001. The covariates were evaluated on the relative bioavailability of lumefantrine and artemether and on elimination clearance for all investigated drugs. The model was thereafter tested for additional covariates and HIV drug effects using the stepwise approach. The investigated covariates were body weight, body mass index, age, which study and gender.

Model diagnostics were performed using goodness-of-fit plots and visual predictive checks (prediction corrected; 2000 simulations) [48]. The final model was bootstrapped (*n* = 1000) to calculate relative standard errors (RSEs) of model parameters and non-parametric confidence intervals around these estimates. η and ε shrinkage were calculated in nonmem.

Stochastic simulations (1000 simulations) were performed using the final models for artemether/dihydroartemisinin and lumefantrine/desbutyl-lumefantrine to investigate the putative drug exposure for new antimalarial dosing regimens when co-administered with HIV drugs. Dihydroartemisinin is more potent than lumefantrine and kills the majority of the parasite bio-mass but is rapidly eliminated from the body (terminal half-life of 1–2 h). Lumefantrine has a longer terminal half-life of 3.3 days and kills the residual parasites that remain in the blood after dihydroartemisinin has been eliminated from the body (i.e. beyond 72 h after the first dose), and this prevents recrudescent malaria. Exposures to lumefantrine (area under the concentration–time curve from 72 h to infinity) and dihydroartemisinin (0 h to infinity) were therefore considered the main endpoints for dose optimization simulations. Day 7 concentrations of lumefantrine were also evaluated since this endpoint is commonly measured and reported in the literature. Exposures were simulated when artemether-lumefantrine was administered alone, when administrated together with the antiretroviral drugs and after an increased dose regimen together with the antiretroviral treatment (based on whole tablet alterations). Dose limited absorption of lumefantrine was taken into consideration by assuming that the exposure only increased 70% for a 100% dose increase [13].

## Results

A total of 89 HIV-infected adult patients without malaria were recruited to two studies. The first (study 1, *n* = 31) was a parallel group study, the second (study 2, *n* = 58) was a crossover study [35,36].

The treatments were well tolerated in both studies. Demographic data are presented in Table 1.

**Table 1:** Demographic data of study population

	Study 1	Study 2
	Median (range)	Median (range)
**Number of patients**	31	58
**Age (years)**	36.5 (24–51)	36 (20–70)
**% females**	61.3	79.3
**Body weight (kg)**	64 (45–86)	56 (42–91)
**Body mass index (kg m^−2^)**	23.7 (17.0–34.0)	22.3 (17.3–36.5)

Study 1 was a parallel study with two arms. Study 2 was a crossover study with three phases.

### Lumefantrine and desbutyl-lumefantrine pharmacokinetics

Data collected in the two clinical studies were pooled and analyzed simultaneously using non-linear mixed effect modelling. A total of 1365 densely collected plasma samples were analyzed for lumefantrine and a sub-set (341 samples, due to a later analysis) for desbutyl-lumefantrine concentrations. Only 99 (7.3%) of the lumefantrine samples and 37 (11%) of the desbutyl-lumefantrine samples had concentrations below the lower limit of quantification (25 ng ml^−1^ and 1.0 ng ml^−1^, respectively) and were therefore omitted. Parent and metabolite kinetics were included in the same model assuming full conversion of the parent drug to its metabolite. A two compartment disposition model was superior to a one compartment model in describing lumefantrine pharmacokinetics (*P* < 0.001). A third distribution compartment did not improve the model fit significantly (*P* > 0.05). Desbutyl-lumefantrine pharmacokinetics were described by a one compartment disposition model. Additional distribution compartments improved the fit significantly but were highly unstable and did not minimize successfully. A transit compartment model with a fixed number of one transit compartment (*k*_a_ and *k*_tr_ assumed to be equal) described the absorption phase better than all other absorption models. A relative bioavailability parameter was implemented to allow for quantification of the between subject variability in the absorption of lumefantrine and resulted in a significant improvement in the model fit (*P* < 0.001). Box–Cox transformation of pharmacokinetic parameters did not improve the fit. The implementation of lopinavir/ritonavir, nevirapine and efavirenz as categorical covariates significantly improved the model (*P* < 0.001). Concomitant administration of lopinavir/ritonavir decreased the elimination clearance of lumefantrine by 62% and increased clearance of desbutyl-lumefantrine by 392%. Concomitant administration of nevirapine lowered the relative bioavailability of lumefantrine by 25% while concomitant administration of efavirenz increased elimination clearance of lumefantrine by 72.6%. No other covariates (i.e. drug interaction effect on other parameters, study, body weight, body mass index, gender or age) had any significant effects.

**Figure 1:**
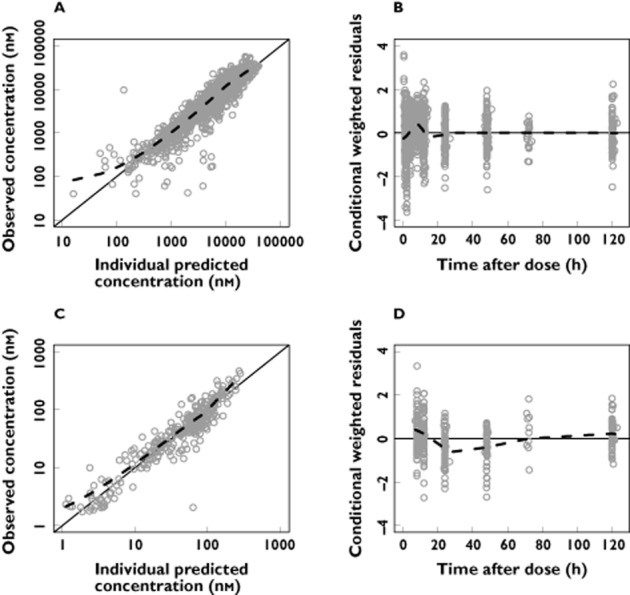
Basic goodness-of-fit plots for the final lumefantrine/desbutyl-lumefantrine model. Observations plotted against individual predicted concentrations of lumefantrine (A) and desbutyl-lumefantrine (C). Conditional weighted residuals of lumefantrine (B) and desbutyl-lumefantrine (D) plotted against time after dose. The solid line is the identity line and the broken line is the locally weighted least square regression line. The concentrations are presented on a logarithmic (base 10) axis

**Figure 2:**
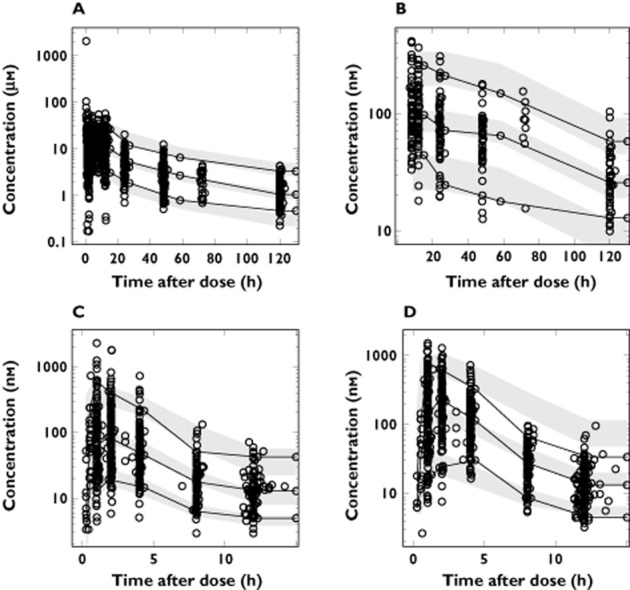
Visual predictive checks of the final models for lumefantrine (A), desbutyl-lumefantrine (B), artemether (C), and dihydroartemisinin (D), based on 2000 simulations. Open circles represent the observations and solid lines represent the 5th, 50th and 95th percentiles of the observed data. The shaded areas represent the 95% confidence intervals around the simulated 5th, 50th and 95th percentiles. The concentrations are presented on a logarithmic (base 10) axis

Between subject variability was retained in the estimates of elimination clearance, the mean transit time and the relative bioavailability of lumefantrine. The goodness-of-fit plots show a good description of the data (Figure 1). Parameter estimates were reliable with small RSEs (Table 2), except for the mean absorption transit time which displayed a high RSE (106%) as few samples were collected during the absorption phase. Calculated η-shrinkages were high for lumefantrine elimination clearance and mean transit time (40% and 47%, respectively) but the calculated ε-shrinkages were low (4% and 6% for lumefantrine and desbutyl-lumefantrine, respectively). Visual predictive checks demonstrated good predictive performance of the final pharmacokinetic model (Figure 2).

**Table 2:** Parameter estimates describing the population pharmacokinetics of lumefantrine and desbutyl-lumefantrine

	Population estimates* [RSE %†]	95% CI†	BSV* [RSE %†]	95% CI†	Shrinkage* (%)
***Lumefantrine***					
**CL/*F* (l h^−1^)**	4.77 [5.30]	4.31, 5.30	14.8 [50.7]	5.68, 22.7	40.1
***V*_c_/*F* (l)**	68.9 [27.1]	47.4, 117	–	–	–
**Q/*F* (l h^−1^)**	2.86 [19.4]	1.72, 3.62	–	–	–
***V*_P_/*F* (l)**	111 [9.14]	93.9, 132	–	–	–
**Number of trans comp**	1 FIX	–	–	–	–
**MTT (h)**	6.27 [21.2]	3.75, 8.35	31.4 [106]	15.1, 94.6	46.9
**Bioavailability (%)**	1 FIX	–	47.4 [18.3]	38.2, 57.4	6.21
**RUV**	0.566 [7.83]	0.479, 0.643	–	–	4.05
**EFZ_CL/_*_F_* (%)**	72.6 [17.2]	51.5, 100	–	–	–
**LOP_CL/_*_F_* (%)**	−62.1 [8.48]	−72.1, −51.8	–	–	–
**NEV*_F_* (%)**	−24.8 [38.6]	−42.4, −4.66	–	–	–
***Desbutyl-Lumefantrine***					
**CL/*F* (l h^−1^)**	489 [5.98]	435, 554	–	–	–
***V*_c_/*F* (l)**	22 800 [7.93]	19 600, 26 800	–	–	–
**RUV**	0.465 [13.3]	0.357, 0.591	–	–	6.05
**LOP_CL/_*_F_* (%)**	392 [17.6]	239, 488	–	–	–

CL/*F* is the apparent elimination clearance. *V*_c_/*F* is the apparent volume of distribution of the central compartment. Q/*F* is the inter-compartment clearance between the central and the peripheral compartment. *V*_P_/*F* is the apparent volume of distribution of the peripheral compartment. MTT is the mean transit time of the absorption. RUV is the variance of the unexplained residual variability. Number of trans comp is the number of transit compartments used in the absorption model. *F* represents the relative bioavailability. EFZ_CL/_*_F_* and LOP_CL/_*_F_* are the effect on elimination clearance by concomitant treatment with efavirenz and lopinavir/ritonavir, respectively. NEV*_F_* is the effect of concomitant treatment with nevirapine on the relative bioavailability. Coefficients of variation (%CV) for between-subject variability (BSV) were calculated as 100 × (e^variance^ – 1)^1/2^. Relative standard errors (RSE) were presented as 100 × (standard deviation/mean). The 95% confidence intervals (CI) are given as the 2.5 to 97.5 percentiles of bootstrap estimates.

* Based on population mean values from nonmem.

† Based on 866 successful stratified bootstrap runs (out of 1000).

### Artemether and dihydroartemisinin pharmacokinetics

In total, 934 plasma samples were analyzed for artemether and dihydroartemisinin concentrations. Of these samples, 277 (30%) and 181 (19%) contained concentrations of artemether and dihydroartemisinin, respectively, below the lower limit of quantification (1.4 ng ml^−1^ for both compounds) and were omitted from the analysis. The M3 method [45] was attempted but unrealistic parameter estimates were obtained. Simulation-based diagnostics of the final model showed no model mis-specification by omitting data below the limit of quantification (results not shown).

Artemether and dihydroartemisinin were modelled simultaneously, assuming full *in vivo* conversion of artemether to dihydroartemisinin. Both drugs were described satisfactorily by one compartment disposition models. For artemether, a two compartment model improved the model fit significantly (*P* < 0.001) but resulted in unrealistic parameter estimates and a highly unstable model. For dihydroartemisinin, a two compartment model did not improve the fit significantly (*P* > 0.05). A transit compartment model (*n* = 3) was superior to all other absorption models (*k*_a_ and *k*_tr_ assumed to be equal). The addition of a relative bioavailability parameter with an estimated between subject variability improved the fit of the model significantly (*P* < 0.001). Box–Cox transformation of the elimination clearance of artemether improved the model fit but resulted in low precision of the transformation factor (RSE = 55%) and decreased precision of the between subject variability of the elimination clearance of dihydroartemisinin (RSE = 168%), and was therefore not retained in the final model.

The model was significantly improved by incorporating metabolic auto-induction of the demethylation of artemether on its first pass liver extraction as investigated in a sub-set of data.

Concomitant administration of lopinavir/ritonavir significantly increased the elimination clearance of both artemether (32.8%) and dihydroartemisinin (143%). Concomitant administration of nevirapine decreased the elimination clearance of dihydroartemisinin by 44% and concomitant administration of efavirenz and nevirapine also resulted in a decreased relative bioavailability of artemether (71% and 66%, respectively). No other covariates had any significant effects.

**Figure 3:**
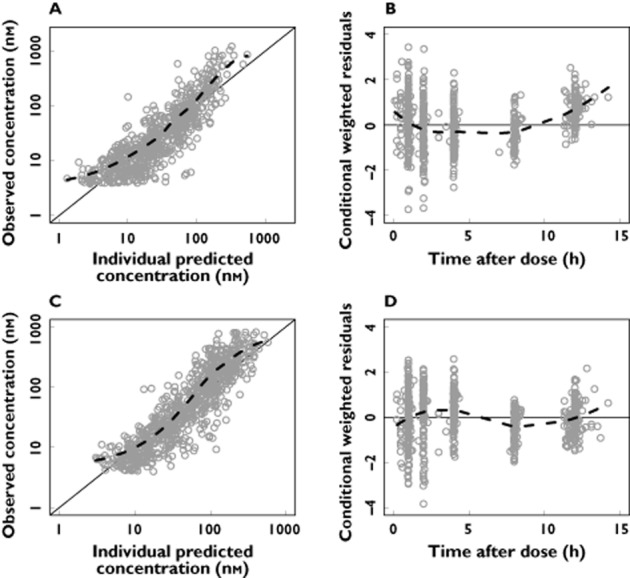
Basic goodness-of-fit plots for the artemether/dihydroartemisinin model. Observations plotted against individual predicted concentrations of artemether (A) and dihydroartemisinin (C). Conditional weighted residuals of artemether (B) and dihydroartemisinin (D) plotted against time after dose. The solid line is the identity line and the broken line is the locally weighted least square regression line. The concentrations are presented on a logarithmic (base 10) axis

Between subject variability was retained in the estimates of elimination clearance of artemether, elimination clearance of dihydroartemisinin, mean transit time and relative bioavailability. Goodness-of-fit plots showed an expected trend of model mis-specification at low concentrations due to censoring of data below the limit of quantification (Figure 3). Calculated η-shrinkage for artemether elimination clearance was high (46%) but calculated ε-shrinkages were low (7% and 8% for artemether and dihydroartemisinin, respectively). The parameter estimates were deemed to be reliable with small RSEs (Table 3). Visual and numerical predictive checks demonstrated good predictive performance of the final pharmacokinetic model (Figure 2).

**Table 3:** Parameter estimates describing the population pharmacokinetics of artemether and dihydroartemisinin

	Population estimates* [RSE %†]	95% CI†	BSV* [RSE %†]	95% CI†	Shrinkage* (%)
***Artemether***					
**CL/*F* (l h^−1^)**	317 [8.25]	270, 374	9.8 [60.6]	2.75, 16.1	50.6
***V*_c_/*F* (l)**	1090 [8.69]	917, 1291	–		–
**Number of trans comp**	3 FIX	–	–	–	–
**MTT (h)**	0.970 [6.58]	0.853, 1.10	51.6 [18.6]	41.1, 61.6	19.0
**Bioavailability (%)**	1 FIX	–	58.6 [27.6]	39.9, 76.6	10.7
**RUV**	0.724 [5.60]	0.644, 0.803	–	–	7.25
**MMAX**	1 FIX	–	–	–	–
**MF50**	62 FIX	–	–	–	–
**Hill**	0.445 [43.9‡]	–	–	–	–
**LOP_CL/_*_F_* (%)**	32.8 [21.5]	21.0, 47.0	–	–	–
**EFZ*_F_* (%)**	−71.5 [5.94]	−79.3, −62.0	–	–	–
**NEV*_F_* (%)**	−66.3 [7.41]	−75.3, −55.9	–	–	–
***Dihydroartemisinin***					
**CL/*F* (l h^−1^)**	160 [4.93]	145, 174	53.9 [31.5]	33.4, 70.4	18.7
***V*_c_/*F* (l)**	14.9 [39.8]	4.22, 27.9	–	–	–
**RUV**	0.707 [4.40]	0.645, 0.764	–	–	8.28
**LOP_CL/_*_F_* (%)**	143 [19.7]	96.2, 207	–	–	–
**NEV_CL/_*_F_* (%)**	−44.5 [14.5]	−56.6, −31.2	–	–	–

CL/*F* is the apparent elimination clearance. *V*_c_/*F* is the apparent volume of distribution of the central compartment. MTT is the mean transit time of the absorption phase. RUV is the variance of the unexplained residual variability. Number of trans comp is the number of transit compartments used in the absorption model. MMAX is the maximum maturation in the maturation model. MF50 is the time in which the maturation has reached 50%. Hill is the Hill coefficient in the maturation model. LOP_CL/_*_F_* and NEV_CL/_*_F_* are the effect on elimination clearance by concomitant treatment with lopinavir/ritonavir or nevirapine, respectively. EFZ*_F_* and NEV*_F_* are the effect on the bioavailability by concomitant treatment with efavirenz or nevirapine, respectively. Coefficients of variation (%CV) for between-subject variability (BSV) were calculated as 100 × (e^variance^ – 1)^1/2^. Relative standard errors (RSE) were presented as 100 × (standard deviation/mean). The 95% confidence intervals (CI) are given as the 2.5 to 97.5 percentiles of bootstrap estimates.

* Based on population mean values from nonmem.

† Based on 932 successful stratified bootstrap runs (out of 1000).

‡ Based on 1000 separate bootstrap runs with a reduced dataset.

### Dose optimization

**Figure 4:**
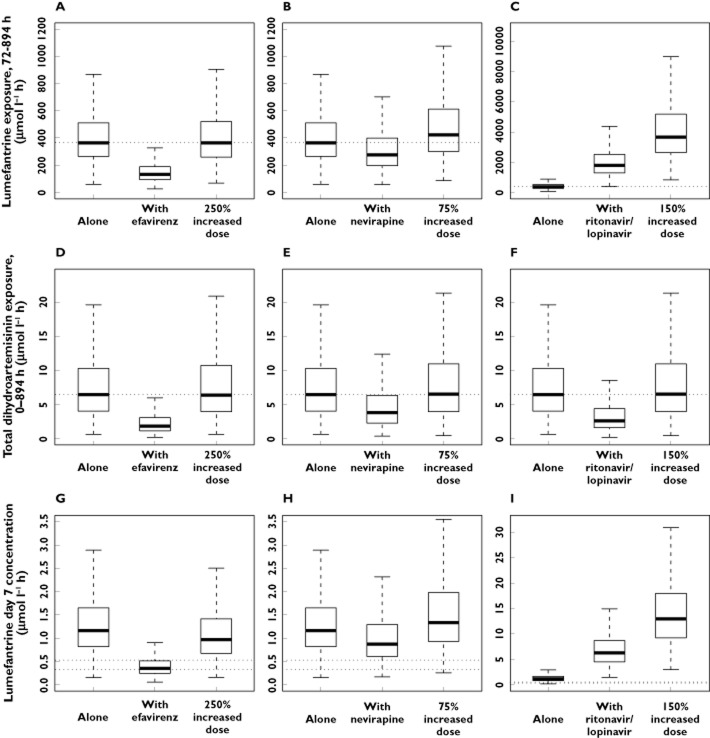
Box (25^th^ to 75^th^ percentile) and whisker (1.5 × interquartile range) plot of dose simulations. The top row illustrates the simulated terminal exposures (AUC) from 72 to 894 h for lumefantrine when given alone, in combination with HIV treatment and after an adjusted dose regimen [efavirenz (A), nevirapine (B) and lopinavir/ritonavir (C)]. The middle row illustrates the simulated exposures (AUC) from 0 to 894 h for dihydroartemisinin when given alone, in combination with HIV treatment and after an adjusted dose regimen [efavirenz (D), nevirapine (E) or lopinavir/ritonavir (F)]. The bottom row illustrates the simulated day 7 concentrations for lumefantrine when given alone, in combination with HIV treatment and after an adjusted dose regimen [efavirenz (G), nevirapine (H) or lopinavir/ritonavir (I)]. The dotted lines in the top and middle rows represent the standard exposures when the antimalarial treatment is given alone. The dotted lines in the bottom row represent previously defined day 7 cut-off concentration for therapeutic failure of 280 ng ml^−1^ and 175 ng ml^−1^

Efavirenz substantially decreased the exposure to both lumefantrine and dihydroartemisinin (decrease of 69.9% and 71.7%, respectively). A 250% increase in dose (assuming a dose dependent bioavailability) would be required to achieve exposures similar to those following a standard artemether-lumefantrine antimalarial treatment without concomitant HIV treatment (Figure 4).

Similarly, nevirapine decreased the exposures to both lumefantrine and dihydroartemisinin (decrease of 25.2% and 41.3%, respectively). A 75% artemether-lumefantrine dose increase (assuming a dose dependent bioavailability) would result in adequate exposures (Figure 4).

Lopinavir/ritonavir increased the exposure to lumefantrine markedly (increase of 439%), but conversely decreased the exposure to dihydroartemisinin (decrease of 59.7%). Thus, because of the fixed dose formulation of artemether-lumefantrine, no dose-adjustment could provide equivalent exposures to those normally achieved. A 150% increase in the artemether-lumefantrine dose would provide dihydroartemisinin exposures similar to those observed in subjects without concomitant antiretroviral treatment (Figure 4), but lumefantrine exposures would then be predicted to be nine times higher. Simulations of lumefantrine day 7 concentrations when given alone, together with antiretroviral treatment and after the new suggested doses are presented in Figure 4.

## Discussion

Drug–drug interactions between artemether-lumefantrine therapy and antiretroviral drugs are likely to have significant implications for the outcome of antimalarial therapy. This is the first study investigating the population pharmacokinetic properties of the antimalarial drugs artemether, dihydroartemisinin, lumefantrine and desbutyl-lumefantrine when co-administered with efavirenz, nevirapine or lopinavir/ritonavir treatment. Previous studies on these interactions have only utilized non-compartmental analysis and/or statistical analysis [31–36]. The presented study uses a non-linear mixed effects approach with a higher statistical power to detect and quantify any interaction. A modelling approach also allows for a mechanistic understanding of the pharmacokinetic properties and their variability. Artemether-lumefantrine is the most widely used artemisinin-based combination treatment in the world so this interaction is likely to be common, and therefore important.

### Pharmacokinetic models

#### Lumefantrine/desbutyl-lumefantrine

In agreement with most previous reports, a two compartment disposition model was selected for lumefantrine, whereas a one compartment model was adequate for desbutyl-lumefantrine [49–51]. A recent publication used a three compartment disposition model for lumefantrine and a two compartment disposition model for desbutyl-lumefantrine [18]. Such a structural model could not be supported by our data, probably because of the shorter follow-up period in our studies. Goodness-of-fit plots and simulation-based diagnostics showed that the model described the data adequately. The parameter estimates were in general agreement with previously published data on elimination clearance and volume of distribution [14,51]. The parameter estimates of desbutyl-lumefantrine were different from those previously published [18], although this may also be a consequence of a shorter follow-up period in our studies and consequently fewer data.

#### Artemether/dihydroartemisinin

Artemether and dihydroartemisinin pharmacokinetics were described by one compartment disposition models. A two compartment disposition model for artemether improved the fit significantly but resulted in high RSEs (>79%) and unrealistic parameter estimates (e.g. half-life of 12.2 h). The most probable explanation of the improved model fit is the large fraction of data below the limit of quantification. The M3 method would be the preferred approach to handle such data but resulted in unreasonable parameter estimates (e.g. half-life of 1020 h) [45]. A one compartment disposition model was therefore considered as the final model and resulted in acceptable goodness-of-fit diagnostics and reasonable predictive performance. Artemether and dihydroartemisinin parameter estimates were in line with previous data from healthy volunteers [5,52].

### Drug–drug interactions

#### Lumefantrine/desbutyl-lumefantrine

Exposure to lumefantrine was decreased substantially by concomitant treatment with efavirenz or nevirapine. This is most likely a result of increased expression of CYP3A4 increasing hepatic extraction in the case of efavirenz and a decrease in bioavailability, possibly due to induction of intestinal P-glycoprotein expression or induction of intestinal CYP3A4, in the case of nevirapine. The exposure to desbutyl-lumefantrine was also decreased by concomitant treatment with nevirapine, due to the lower bioavailability of lumefantrine.

In contrast, lopinavir/ritonavir increased the exposure to lumefantrine. Lopinavir/ritonavir inhibits CYP3A4 activity [27], resulting in a decreased elimination clearance of lumefantrine and consequently an increased exposure. However, elimination clearance of desbutyl-lumefantrine was increased by lopinavir/ritonavir resulting in decreased exposure to desbutyl-lumefantrine. The mechanism of elimination for desbutyl-lumefantrine is not known, and therefore the effect of lopinavir/ritonavir could be a result of changes in the elimination of desbutyl-lumefantrine.

These results are comparable with the results of the model-independent non-compartmental analyses of these studies [35,36]. The incorporated covariates in the present study also identified an additional trend of lowered lumefantrine exposure when combined with nevirapine.

#### Artemether/dihydroartemisinin

All three antiretroviral drugs investigated decreased the exposures to both artemether and dihydroartemisinin. Efavirenz and nevirapine decreased the relative bioavailability of artemether, which would explain the decreased exposure of the drug and metabolite. The available findings on the interaction between efavirenz/nevirapine and P-glycoprotein are contradictory. An *in vitro* study showed that efavirenz and nevirapine induce the expression of P-glycoprotein [26] which could explain the observed results. Another explanation could be induction of intestinal CYP3A4 enzymes although this would preferentially affect artemether.

Nevirapine also decreased the elimination clearance of dihydroartemisinin, but not to the same extent as the decrease in bioavailability of artemether, resulting in a total decrease in the exposure to dihydroartemisinin. The decrease in dihydroartemisinin elimination clearance in the present study is difficult to explain since nevirapine has not been reported to affect the UGT-system. The present study showed similar results compared with earlier studies with the exception of the study by Kredo *et al*. (in which they, in contrast, present a trend of increased exposure) [33]. This might be explained by between subject variability, different study sizes (36 compared with 89 in this study) and different study designs (parallel compared with crossover in this study).

Lopinavir/ritonavir increased the elimination clearance of both artemether and dihydroartemisinin resulting in decreased exposures. Lopinavir/ritonavir induces other CYP enzymes such as CYP2B6, CYP2C9 and CYP2C19. Induction of CYP enzymes seems the most likely explanation for the observed increase in the elimination clearance of artemether. The increase in dihydroartemisinin elimination clearance is unexpected since lopinavir/ritonavir inhibits several UGT enzymes which would be expected to result in a decreased clearance. The increased clearance is so far unexplained but it could be a consequence of lopinavir dependent induction of other unknown metabolic pathways of artemether.

The results for artemether-dihydroartemisinin are also similar to the results of the previously published non-compartmental analyses [35,36] except that the modelling approach also identifies a decreased dihydroartemisinin exposure when combined with lopinavir/ritonavir which was only indicated as a trend in the non-compartmental analyses.

### Dose optimization

Artemether-lumefantrine is provided as a fixed dose combination tablet. The simulations aimed to investigate how changing dosing (the normal adult dose being four tablets per dose twice daily for 3 days), would influence the systemic exposure to the antimalarial compounds. The resulting recommendations are tentative. This study was based on HIV-infected subjects with no evidence of malaria infection. Malaria itself, depending on clinical severity, may affect drug absorption, distribution and elimination and may therefore influence the degree of the interactions described here. Determining the clinical relevance of these observations will require further study in patients on antiretroviral treatment who have acute malaria.

If it is assumed that a malaria infection does not mitigate the drug–drug interactions, and there was a linear relationship between dose and exposure then an increase in artemether-lumefantrine dose of 250% would therefore be required for patients on efavirenz treatment. As single adult doses over four tablets may not be absorbed adequately, dose increases would necessitate increased frequency of dosing or a longer treatment course. The most reliable approach to increasing exposure would be to use a 7 day regimen. Whether higher individual doses or increased frequency (three or four times daily) would allow shorter courses needs to be determined prospectively.

Concomitant treatment of lopinavir/ritonavir resulted in decreased dihydroartemisinin exposure. This might result in a smaller fraction of parasites being killed during the first 72 h of treatment. This potentially reduced pharmacodynamic effect is balanced by the concomitant marked increase in exposure to lumefantrine. In addition, the possible weak antimalarial effect of the protease inhibitor might contribute to therapeutic efficacy [31]. Whether efficacy would be increased or decreased in low transmission settings is uncertain.

The predicted increase in artemether-lumefantrine dose in HIV patients treated with nevirapine is 75%. As for efavirenz the dosing regimen would require a prospective study to determine whether this regimen could still be given over 3 days.

The simulated day 7 concentrations of lumefantrine exhibit a similar trend compared with lumefantrine exposure (area under the concentration–time curve, Figure 4). Two previous studies have reported that a day 7 concentration below 280 ng ml^−1^ and 175 ng ml^−1^, respectively, will result in an increased risk of treatment failure [51,53]. The mean lumefantrine day 7 concentrations when combined with efavirenz are below 280 ng ml^−1^, but close to 175 ng ml^−1^. The new proposed dose regimen increases the mean day 7 concentration above 280 ng ml^−1^. When combined with nevirapine the mean day 7 concentration will decrease but not fall below 280 ng ml^−1^ and a combination with ritonavir boosted lopinavir will increase the mean day 7 concentration. This suggests that the lower exposure after concomitant administration of nevirapine would be of less clinical importance. This also emphasizes the need to investigate this in a population infected with malaria.

In conclusion, this study evaluated the impact of concomitant HIV treatment with efavirenz, nevirapine or lopinavir/ritonavir based regimens on the pharmacokinetic properties of artemether, lumefantrine and their respective metabolites. All HIV drugs influenced the exposure to artemether-lumefantrine which was attributed to several changes in the pharmacokinetics of the antimalarial drugs. Efavirenz and nevirapine decreased the exposure to lumefantrine and dihydroartemisinin and dose adjustments may be required. The exposure to lumefantrine was increased when combined with lopinavir/ritonavir although in this case a change in dose might not be required. The proposed new dose regimens for the artemether-lumefantrine fixed combination do not take the effect of malaria infections on pharmacokinetics into consideration. This needs to be evaluated in clinical studies in malaria-HIV co-infected patients.
